# Use of Ultrasound to Diagnose and Manage a Five-Liter Empyema in a Rural Clinic in Sierra Leone

**DOI:** 10.1155/2014/173810

**Published:** 2014-06-22

**Authors:** Masashi Rotte, Jason Matthew Fields, Sergio Torres, Christa Dominick, J. Daniel Kelly

**Affiliations:** ^1^Department of Emergency Medicine, Thomas Jefferson Hospital, Room 239, 1020 Sansom Street, Philadelphia, PA 19107, USA; ^2^Department of Internal Medicine, Baylor College of Medicine, Suite 1100.D, 6620 Main Street, BCM 620, Houston, TX 77030, USA; ^3^Jefferson Medical College, Room 239, 1020 Sansom Street, Philadelphia, PA 19107, USA; ^4^Wellbody Alliance, 100 Doma Street, Koidu, Kono District, Sierra Leone; ^5^Division of Infectious Diseases, Department of Medicine, University of California, 513 Parnassus Avenue, S-380, San Francisco, CA 94131, USA

## Abstract

We report the case of a dyspneic patient with a five-liter pleural empyema that was diagnosed and managed in a resource-limited clinic in a rural part of Sierra Leone. The diagnosis and management of this condition are usually guided by imaging modalities such as X-rays or CT scans. However, these resources may not be available in austere settings in developing countries. Because emergency physicians work in a variety of clinical settings, they should be well versed in the use of portable ultrasound machines to diagnose, treat, and manage many different conditions.

## 1. Introduction

Many regions of the world suffer from limited resources, including lack of medical imaging, preventing effective diagnosis and treatment of many conditions. Due to its decreasing cost and size, portable ultrasound has become a potential solution for this problem and many emergency physicians have helped to implement ultrasound programs in austere environments [1–3]. We report the case of a young and otherwise healthy man who had a five-liter empyema diagnosed and managed solely with the use of portable ultrasound in a resource-limited setting in Sierra Leone.

## 2. Case Presentation

A 28-year-old male presented to a clinic in a rural part of Sierra Leone with a chief complaint of dyspnea. His symptoms began 23 days earlier with a cough productive of yellow sputum, myalgias, fever, and fatigue. After one week of symptoms, the sputum became bloody and the patient noticed that he was losing weight. The patient then sought care from a traditional healer near his village who prescribed a drink made of local leaves as well as two unknown medications. Over the next two weeks, the patient's condition worsened so he traveled to the clinic.

The patient was born in Sierra Leone and had never left the country. He denied any medical, surgical, or relevant family history and took no medications regularly. The patient worked as a gold miner, smoked 1 pack of cigarettes per day for 10 years, and denied alcohol or drug use. Review of systems was otherwise unremarkable.

Initial examination revealed an acutely dyspneic and diaphoretic male using accessory muscles to breathe and tripoding. Vital signs revealed tachypnea with respiration of 40/min, pulse of 102 beats/min, axillary temperature of 37.2°C (98.9°F), blood pressure of 120/85 mmHg, and pulse oximetry of 94% in room air. The lung exam revealed decreased breath sounds, dullness to percussion, and decreased tactile fremitus of the entire right thorax with midline trachea. Cardiac exam demonstrated tachycardia with a regular rhythm, normal S1 and S2, and no jugular venous distension. The sclera and mucous membranes were anicteric and the abdominal exam revealed only a freely reducible umbilical hernia but no organomegaly. No rashes or erythema was noted on the skin and no peripheral edema was found.

**Figure 1:**
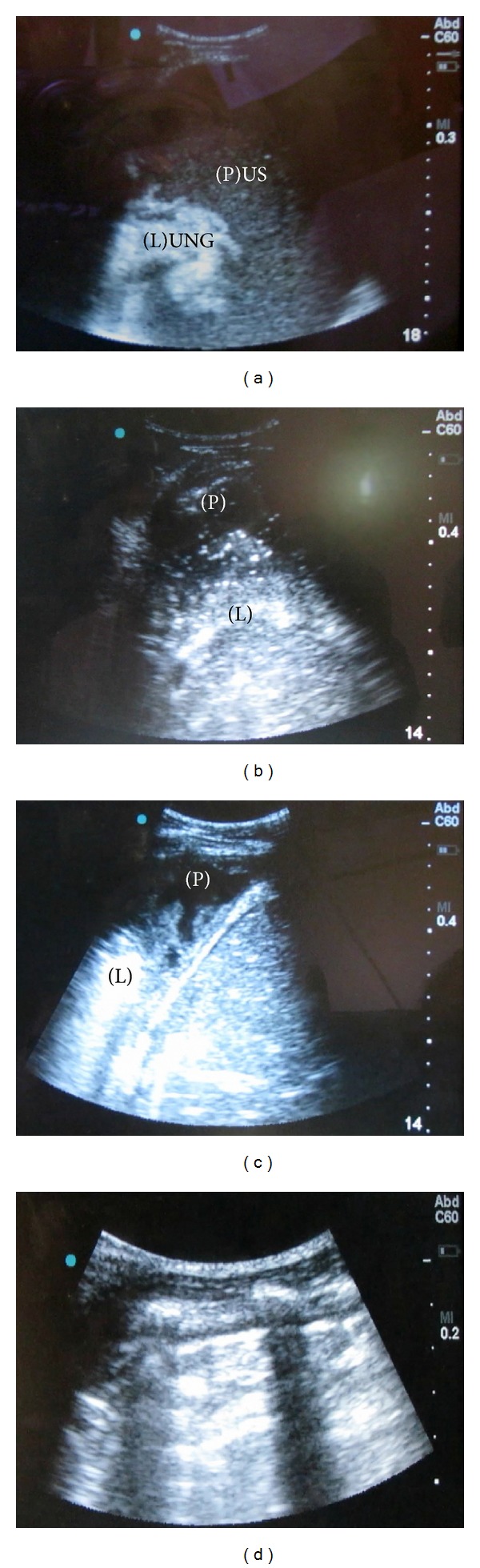
(a) The initial ultrasound demonstrating a large amount of fluid in the pleural space and a collapsed lung. (b) An ultrasound one day after the drain was placed demonstrating decreased fluid in the pleural space. (c) An ultrasound three days after the drain was placed demonstrating even less fluid and improved lung expansion. (d) An absent sliding sign on the affected hemithorax.

**Figure 2:**
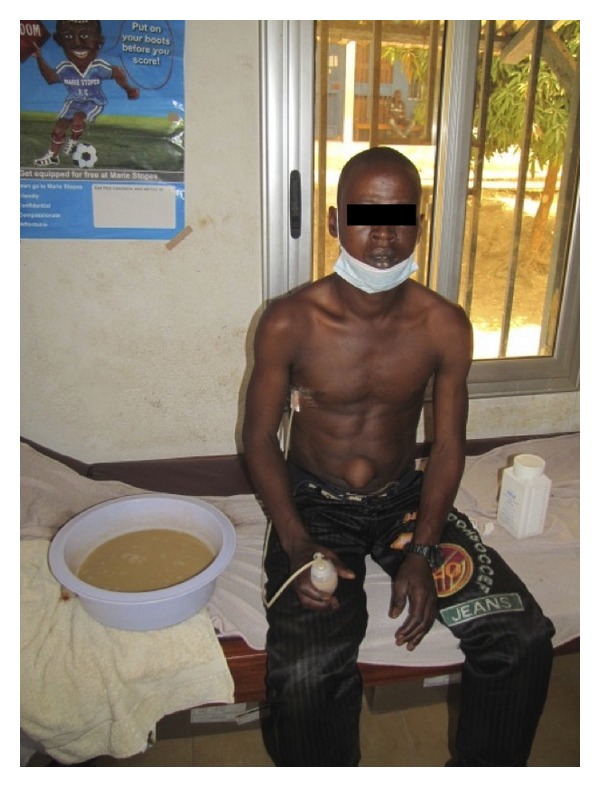
The patient after the JP drain was placed sitting next to approximately one-half of the empyema volume that was removed.

As no X-ray or CT scan machines were available in the clinic, an emergent bedside ultrasound was performed using a Titan ultrasound machine with a C60x/5-2 MHz curvilinear probe (Sonosite, Inc., Bothell, WA 98021, USA). A large pleural effusion was noted in the right hemithorax with collapsed and free-floating lung (Figure 1(a)). No septation was noted. As medical supplies were limited in the austere setting of the clinic, a Jackson-Pratt (JP) drain was placed through a thoracostomy in the right midaxillary line using Kelly forceps. No thoracostomy tubes or suction machines were available. Upon placement of the drain, there was immediate return of moderately thick and purulent fluid. During the next hour, over 5 liters of purulent fluid was drained and the patient's respiratory distress rapidly improved (Figure 2).

The patient was monitored and treated with intravenous ceftriaxone and metronidazole. The patient had several laboratory tests performed while he was at the clinic. He had a nonreactive HIV test and the pleural fluid revealed gram-positive cocci in clusters and chains and gram-negative rods. A Kinyoun stain was negative for acid fast bacilli. Additional tests such as pH, protein, or culture for organism identification and drug susceptibility testing were not available at the clinic.

After drainage, serial ultrasounds were conducted on day 1 (Figure 1(b)) and day 3 (Figure 1(c)) and showed decreased fluid in the pleural space, improved lung expansion, and a small pneumothorax (Figure 1(d)) diagnosed by the lack of a positive sliding sign. Smaller volumes of purulent fluid were recorded from the JP drain on a daily basis. After day 5, the JP drain output stopped so the drain was removed and the patient was monitored in the clinic for two more days. An improvised incentive spirometer was made from an empty saline bottle and the patient was instructed on deep breathing exercises. After day 7, the patient was discharged home in stable condition with a course of oral antibiotics and precautions. At one-year follow-up, the patient was in good health.

## 3. Discussion

Empyema thoracis is defined as “pus in the thoracic cavity due to a pleural space infection” and has been described since the 5th century [4]. Our patient's clinical presentation was akin to the natural history of an untreated pneumonia, which in the pre-antibiotic era commonly resulted in an empyema. Three progressive stages of empyema have been described. The first is an exudative stage with free-flowing pus in the pleural space. The next is a fibrinopurulent stage in which fibrin deposits lead to septation and loculations. Finally, in the organized stage, the septation and loculations thicken the pleura and trap the lung. Emypema usually results from pneumonia, including pneumonia due to tuberculosis, but can also be caused by trauma, iatrogenically from thoracic procedures, hematogenous spread, or extension from nearby infections. Risk factors for an empyema include immunocompromise, alcohol or intravenous drug abuse, diabetes, gastroesophageal reflux disease, poor dental hygiene, chronic lung disease, and rheumatoid arthritis [5].

Worldwide, the incidence of pleural space infections has been increasing likely due to the spread of HIV and tuberculosis [4]. The initial clinical presentation of an empyema is not dissimilar to that of pneumonia and the diagnosis requires a high index of suspicion. Patients may complain of fever, cough, chest pain, shortness of breath, or weight loss. Based on risk factors and clinical context, providers may be more likely to consider empyema in their differential diagnosis. When the patient is an immunocompromised host, however, providers should have a heightened awareness that sick patients tend to appear well and clinical presentations follow a more sub-acute course [6].

Imaging studies are important diagnostic aids when evaluating a patient suspected of having an empyema. Empyema should be differentiated from a pulmonary abscess, which is contained in the lung parenchyma, and a pleural effusion, which does not contain pus [6]. Chest radiography should be the initial imaging study; it may show fluid levels and pulmonary infiltrates. Computed tomography of the thorax can also be a valuable study in characterizing the lung process and differentiating pathologies (i.e., empyema and lung abscess) [7]. In some clinical settings, such as the clinic in Sierra Leone, neither of these imaging modalities is available. Fortunately, ultrasound has been used to diagnose pleural disease since the 1960s [8]. Ultrasound has many benefits over other imaging modalities: it does not expose the patient to radiation, it is portable and can be performed at the bedside, many modern machines run on batteries, thus obviating the need for a reliable source of electricity, and the only additional supply needed is ultrasound gel (which can be made from cornstarch) [9]. Ultrasound can also be used therapeutically to guide needle thoracentesis, to determine the ideal intercostal space for tube thoracostomy, to identify septation or loculations potentially indicating a more complicated course [10], and to follow the volume of empyema remaining in the thoracic cavity after a drain is placed.

The current mainstays of treatment for empyema are evacuation of the purulent fluid, chest physiotherapy, and appropriate antibiotics [11]. Empyema evacuation has been successfully performed with small gauge pigtail catheters and larger gauge chest tubes. Empyemas in more advanced stages may require more invasive procedures such as intrapleural fibrinolytics, thoracoscopy, video-assisted thoracic surgery (VATS), or fenestration; in the most advanced or refractory cases, open surgery may be required [5, 12]. These more invasive procedures may not be feasible in resource-limited settings. If laboratory facilities are available, pus drained from an empyema should be sent for chemistry and microbiological studies [6]. Chest physiotherapy may consist of deep breathing exercises, the use of an incentive spirometer, patient turning, and directed coughing [13].

Antibiotic selection should be guided by blood and pleural fluid cultures and Gram stain; these studies may not be available in austere settings. Initial therapy should be intravenous and empirically broad spectrum to cover organisms relevant to the patient's risk factors. Selection of an empiric anti-microbial regimen should be made with consideration to the mechanism of infection. Even though untreated or inappropriately treated pneumonias tend to form a monomicrobial empyema, aspiration events introduce a poly-microbial infection into the lungs. Until the infectious pathogens can be isolated, it is reasonable to initiate anti-microbial coverage for oral flora, which includes gram-positive, gram-negative, and anaerobic organisms. [5]. A three-week course of antibiotics is recommended, but antimicrobial therapy should be not stopped until the empyema demonstrates radiographic resolution [7]. The main pitfall in the management of empyema is due to incomplete drainage of the purulent fluid due to suboptimal placement of the drain, clogging or kinking of the drain, or loculations that prevent complete evacuation with a single drain [11]. Multiple studies have shown the utility of ultrasound to diagnose and treat empyemas [14–16].

To our knowledge, this is the first case of an empyema diagnosed and managed solely by portable ultrasound. Further, this case demonstrates the importance and utility of portable ultrasound especially in resource-limited settings where no other diagnostic medical imaging existed. Emergency physicians can be faced with critical illness in developing countries [17], and we hope to demonstrate the resourcefulness of ultrasound for these settings. Building the human resource capacity of ultrasound care in developing countries can address gaps in care and potentially save lives [18].
